# ApoE Mimetic Peptides to Improve the Vicious Cycle of Malnutrition and Enteric Infections by Targeting the Intestinal and Blood-Brain Barriers

**DOI:** 10.3390/pharmaceutics15041086

**Published:** 2023-03-28

**Authors:** Reinaldo B. Oriá, Raul S. Freitas, Cássia R. Roque, José Carlos R. Nascimento, Ana Paula Silva, João O. Malva, Richard L. Guerrant, Michael P. Vitek

**Affiliations:** 1 Laboratory of Tissue Healing, Ontogeny and Nutrition, Department of Morphology, School of Medicine, Institute of Biomedicine, Federal University of Ceara, Fortaleza 60430-270, Brazil; 2 Institute of Health Sciences, Medicine, University of International Integration of Afro-Brazilian Lusofonia, Redenção 62790-970, Brazil; 3 Institute of Pharmacology and Experimental Therapeutics and Institute for Clinical and Biomedical Research (iCBR), Faculty of Medicine and Center for Innovative Biomedicine and Biotechnology (CIBB), University of Coimbra, 3000-548 Coimbra, Portugal; 4 Division of Infectious Diseases and International Health, Department of Medicine, School of Medicine, University of Virginia, Charlottesville, VA 22908, USA; 5 Division of Neurology, Duke University Medical Center, Durham, NC 27710, USA

**Keywords:** apoE mimetic peptides, environmental enteric dysfunction, blood-brain barrier, gut-brain axis, intestinal inflammation, malnutrition

## Abstract

Apolipoprotein E (apoE) mimetic peptides are engineered fragments of the native apoE protein’s LDL-receptor binding site that improve the outcomes following a brain injury and intestinal inflammation in a variety of models. The vicious cycle of enteric infections and malnutrition is closely related to environmental-driven enteric dysfunction early in life, and such chronic inflammatory conditions may blunt the developmental trajectories of children with worrisome and often irreversible physical and cognitive faltering. This window of time for microbiota maturation and brain plasticity is key to protecting cognitive domains, brain health, and achieving optimal/full developmental potential. This review summarizes the potential role of promising apoE mimetic peptides to improve the function of the gut-brain axis, including targeting the blood-brain barrier in children afflicted with malnutrition and enteric infections.

## 1. Introduction

Apolipoprotein E research has been ignited by breakthrough protein-target engineering to deliver novel apoE mimetic peptides with neuroprotective functions in different models of brain neuroinflammation. These models range from in vitro to in vivo studies of neurodegenerative and neurovascular diseases. In addition, recent studies have associated novel apoE mimetic peptides with improvements in intestinal barrier function, reductions of intestinal inflammation, and hastening of intestinal mucosal recovery after experimental challenges with gut-toxic/harmful compounds.

Our studies and those of others have highlighted the importance of a healthy gut-brain axis to protect brain development in impoverished areas of the developing world where poor sanitation and hygiene result in environmental-related enteric dysfunction [1,2,3,4,5]. If enteric dysfunction is chronic and persistent, even at a low-grade insult, it could facilitate the mounting of a vicious cycle of malnutrition and enteric infections that can disturb normal developmental trajectories and even cause irreversible physical and cognitive faltering [6,7,8,9].

This review summarizes and updates the literature on apoE mimetic peptides protecting against brain and intestinal inflammation. In addition, this review aims to broaden our understanding of how tropical/environmental enteropathy in children negatively affects their cognitive and physical development as a function of APOE genotypes by disturbing the gut-brain axis. Such insults may cause long-term developmental deficits in growing children when occurring at key times of microbiota and brain development, which could increase the risk for neurodegenerative and metabolic disorders later in life [10,11,12]. The pursuit of novel apoE mimetic peptides that target the intestinal and brain barriers for interrupting this vicious cycle of malnutrition and enteric infections, especially in more genetically predisposed children (thriving under adverse environments), may lead to novel and effective therapies for precision medicine in the field of childhood infectious diseases.

## 2. Vicious Cycle of Malnutrition and Enteric Diseases

### 2.1. Prolonged Malnutrition/Enteric Infections Early in Life as a Cause of Environmental Enteric Dysfunction: Mechanisms and Burdens of Disease

The magnitude of malnutrition and enteric infections in early life for children living in impoverished areas may be enormous and largely unappreciated, with acute and long-term effects during their lifespan [13,14]. Children from undernourished mothers are more prone to generate undernourished offspring. Altered in-utero epigenetic programming of genes regulating immunoinflammatory signaling pathways may predispose the offspring to chronic diseases later in life, such as diabetes and other metabolic disturbances [15,16]. New data have shown that epigenetic modifications may be inherited [17] and thus malnutrition/enteric infections could have critical transgenerational consequences with lasting health impacts. Maternal poverty and low education are risk factors for low birth weight and consequent delays in infant growth in disenfranchised families living in poor sanitary conditions [18,19]. Recurrent low birth weights in children may facilitate the intergenerational vicious cycle of malnutrition that has adverse consequences for children’s survival, growth, and neurodevelopment [19,20,21]. Maternal bowel function during pregnancy is critical for healthy fetal and infant development [22]. The microbiota from undernourished mothers that may be transferred to their offspring during delivery and breastfeeding may also play a role in affecting the intestinal microbiota of children and possibly the intestinal absorption of nutrients and fat [23,24,25,26,27].

Environmental enteric dysfunction (EED), also known as “impoverished gut”, is a chronic and most often subclinical condition that affects the intestinal mucosa, leading to villus blunting and crypt hyperplasia, tight junction epithelial unsealing with reductions in the total intestinal absorptive area, intestinal barrier breakdown, and food/enteric pathogen antigen translocation. EED often occurs in highly contaminated poverty settings where exposures to enteric pathogens prevail, especially in the developing world. Children are often more afflicted by EED, with recurrent episodes of enteric infections and malnutrition [28,29]. Food insecurity, pro-inflammatory and contaminated diets, a lack of potable water, and enteric pathogen exposures aggravate and are aggravated by this condition that may be asymptomatic [28,30]. Early postnatal physical development markers, such as low height-for-age z scores (HAZ), and blood and fecal biomarkers, such as lipopolysaccharide (LPS), LPS-binding protein, serum amyloid A, C-reactive protein, alpha-1-antitrypsin, neopterin, intestinal fatty acid binding protein, and others, are important to identify children in most need for interventions [29,31,32].

The vicious cycle of malnutrition and enteric infections early in life among children is very difficult to overcome, and the United Nations expected milestones appear likely to fail in achieving the Sustainable Development Goals proposed by 2030 around optimal nutrition, calling attention to the importance of a better understanding of EED pathophysiology and outcomes [33,34]. Less invasive biomarkers are of utmost importance for the early diagnosis of EED and its appropriate management. The recent use of new technologies, such as transcriptomic analysis, are already shedding light on novel underlying mechanisms of the disease [35].

Accumulating evidence indicates that gut dysbiosis due to pathogenic and prolonged immature intestinal microbiota caused by repeated exposures to environmentally transmitted pathogens contributes to disturbances in brain development [36,37]. A chronic leaky gut may facilitate increased bacterial translocation from the gut into the intestinal mucosa and even the bloodstream, triggering local as well as systemic inflammation. Circulating blood bacteria and LPS may induce neutrophil and monocyte release of myeloperoxidase (MPO), from their azurophilic granules, leading to a blood-brain barrier disruption, ultimately resulting in brain neuroinflammation. Increased brain MPO, which could also be released by activated microglia, a macrophage-like brain cell, has been linked to stroke and amyloid plaques [38,39]. Unpublished data from our group using a neonatal model of cryptosporidiosis and malnutrition in mice showed increased fecal, serum, and prefrontal cortex levels of MPO, suggesting that abnormally high MPO signaling could be a promising target for intervention to break this gut-brain axis disruption.

Cryptosporidiosis, an enteric disease caused by a protozoan gut trophic parasite, is often seen as other enteric pathogens with or without overt symptoms in undernourished children in their first years of life, causing chronic enteric inflammation with or without diarrhea, especially when children are being weaned from exclusive breastfeeding to contaminated water and food [40,41]. Growth declines, even without diarrhea, are frequently associated with cryptosporidiosis in children in adverse environments. Studies carried out in mice infected with *Cryptosporidium* show worse outcomes when on a protein-deficient diet, which may mimic some of the metabolic alterations observed in children with malnutrition and help elucidate the mechanisms related to physical growth impairments. Interestingly, microbiota-derived trimethylamine N-oxide (TMAO) is increased with cryptosporidiosis under low protein diets and may increase the risk for cardiovascular diseases as well [42]. These seminal findings have provided experimental evidence of the deleterious consequences of early enteric infections and protein deficiency in children living in resource-limited environments, predisposing them toward non-communicable chronic diseases later in life [43].

Malnourished children with cryptosporidiosis living in endemic areas of Haiti and northeastern Brazil had evidence of intestinal or systemic inflammation (from mild to moderate degrees) with findings of elevated fecal and systemic levels of lactoferrin and interleukin-8 (IL-8) with TNF values more significantly increased than those observed in healthy adult volunteers [9,44,45]. Measures to restore the intestinal barrier function and thus reduce intestinal inflammation have the potential to prevent or lessen the subsequent vicious cycle of malnutrition and infection that may persist well into late childhood [11,46].

**Figure 1:**
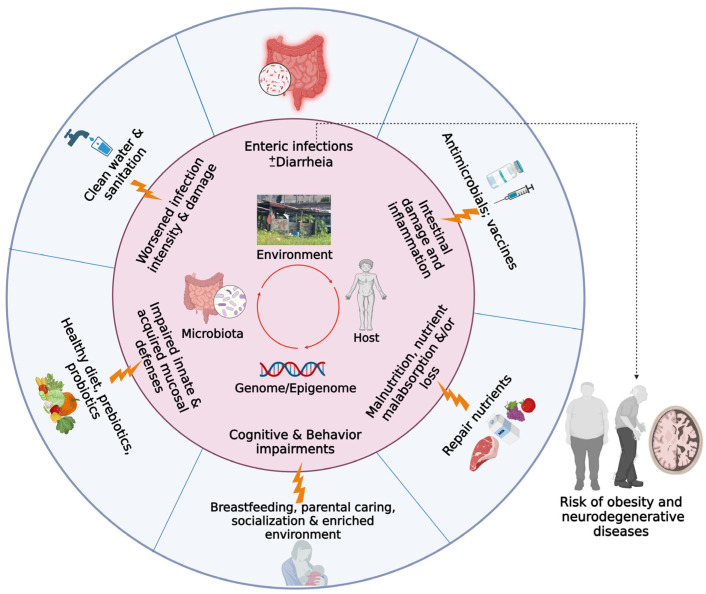
Overview of the vicious cycle of malnutrition and enteric infections (even without diarrhea) early in life. Complex interactions of contaminated environments, gut microbiota dysbiosis, and the hosts genome/epigenome may lead to lasting health effects, including intestinal barrier breakdown and inflammation, poor immune responses against enteric pathogens, and circulating gut born LPS to the brain during critical times of development with cognitive/behavioral impairments. If chronic and persistent, this vicious cycle could increase the risk for cardiovascular and neurodegenerative diseases later in life. In-place continuous interventions, including access to potable water, sanitation, vaccines, and healthy diets, are needed to ameliorate and possibly reverse such deleterious effects. Figure created with BioRender.com.

The causes and consequences of the vicious cycle of malnutrition and enteric illnesses are multifaceted. The long-term deleterious effects of malnutrition in early childhood have been linked to cognitive and physical growth deficits across generations and are believed to suppress immunity to new infections and reduce the effectiveness of vaccines for children. Conversely, the negative effects of enteric infections on cognition may be independent of malnutrition [47]. Figure 1 depicts the vicious cycle of malnutrition and enteric infections and some strategies to ameliorate their lasting health impacts. A multicenter study called MAL-ED (Etiology, Risk Factors, and Interactions of Enteric Infections and Malnutrition and Child Health Consequences Study) was established in 8 countries in the developing world, enrolling children with a high incidence of diarrhea and malnutrition early in life [48,49]. The central objective of MAL-ED was to dissect the short and long-term effects of malnutrition and enteropathies on developmental trajectories and to develop actions to improve the lives of these children in risk areas [50]. A case-control study from MAL-ED in pediatric populations revealed almost ubiquitous enteric infections (even without overt diarrhea), that were often associated with increased intestinal, systemic inflammation, along with growth deficits in children [31].

Kosek and colleagues showed increased intestinal neutrophil activity (MPO), the TH1 immune response (NEO), and protein loss were associated with impaired linear growth in the first few months of life in the MAL-ED children [51]. The significant association of linear growth failure with high levels of gut inflammation markers supports the hypothesis that gut inflammation resulting from frequent enteric infections is an important contributor to faltering childhood growth rates.

EED-related persistent low-grade systemic inflammation may also be a concern for growing children since it may cause anemia of inflammation or anemia of chronic diseases [52,53]. Such conditions may be aggravated by the exploitation of enteric parasites [54,55]. Hepcidin, a liver hormone, is released during systemic inflammation and binds and degrades ferroportin, an iron exporter from cells, thereby reducing available extracellular iron and leading to high levels of intracellular ferritin and poor bone marrow-erythroblast hemoglobin synthesis [56]. Recently, red blood cells have been implicated in innate immunology responses, as these cells express surface TLR9 and may bind to CpG-DNA motifs that may be released through increased intestinal mucosal cell shedding. Red blood cells tagged with TLR9-CpGs are promptly phagocyted by macrophages [57]. Anemia is a growing concern for optimal brain development in children living in adverse environments [58]. Inability to deliver iron to white matter oligodendrocyte precursors may impair myelination in critical brain areas and affect normal cognitive development. Of note, both high-fat diets and enteric pathogens may increase hepcidin levels, which are associated with poor brain myelination in mice [4,59,60].

High rates of enteric dysfunction and diarrhea affect most of the world’s population living in poverty because of their lack of access to basic sanitation and potable drinking water. The close follow-up of this at-risk population by health professionals has led to improved child mortality through oral rehydration therapy, helping to reduce diarrheal burdens. Even with this emergency measure, enteric infections may persist, disrupting intestinal absorption and resulting in barrier functions and sustained growth stunting [11,61,62]. Early diet restrictions and enteric pathogens influence the active remodeling of the intestinal microbiota in the first months of life when the characteristics of the microbiota of adults develop. Children who suffer from malnutrition and enteric disorders in the first years of life may display bacterial overgrowth of the small intestine, leading to low-grade chronic subclinical systemic inflammation [63,64]. Prolonged leaky gut-derived inflammation leads to impaired growth, metabolism, and cognitive development, including declines in IQ, executive and overall memory scores after neuropsychological assessments later in childhood [65,66,67].

Some studies have documented the range of pathogens associated with community diarrhea as well as with growth failure in children in the developing world [68]. The degree of pathogen-specific diarrhea changes depends on the level of pathogen exposure, and high rates of enteric pathogens have been detected even without the occurrence of diarrhea [49].

Lower IGF-1 and higher growth hormone (GH) levels were associated with growth deficits in children. High-sensitivity C-reactive protein serum levels were associated with symptomatic children with enteric infections, diarrhea, and fever. These findings are consistent with an inflammation model leading to hepatic resistance to GH signaling, as observed in preclinical studies [19]. Interestingly, Schwabkey and colleagues have linked mucus-degrading bacteria in the gut to increased fever. These bacteria thin the mucus layer in mice, potentially exposing the hosts to further bacterial infections [69].

### 2.2. Effect of Enteropathy and Gut Dysbiosis on Neurological/Cognitive Outcomes

Enteropathy is often associated with gut dysbiosis. The gut microbiome has been increasingly recognized as a critical player in biological functions and metabolism, affecting children’s brain plasticity. Some intestinal bacterial species, such as *Lactobacillus* and *Bifidobacterium*, can be beneficial for host health and are commonly associated with promoting intestinal barrier integrity by upregulating the expression of tight junction epithelial proteins and reducing the levels of pro-inflammatory cytokines [70].

Any problems that could lead to the reduction or alteration of the normal microbiota may result in a dysbiosis state, causing an imbalance in the gut-brain axis characterized by the disturbance of the blood-brain barrier and reduction of tight junction protein expression [71]. Pathogenic bacteria such as *E. coli* strains, *Salmonella*, *Shigella*, *Helicobacter pylori*, *Vibrio* and *Clostridium* can cause disease by producing a class of exotoxins lipopolysaccharides (LPSs), which damage epithelial cell integrity by targeting the proteolytic degradation of tight junction proteins including the cell adhesion molecule E-cadherin, therefore increasing intestinal permeability [72].

Recently, studies have suggested that dysregulation of the gut microbiome can cause neuroinflammatory disturbances in the CNS that are associated with the progression of neurodegenerative diseases, cognitive impairment, and dementia [73]. Aging, malnutrition, and life in adverse environments can alter the gut microbial profile, which is responsible for increasing the secretion of LPSs and abnormal amyloid proteins aggravating the pathogenesis of Alzheimer’s disease (AD) and others [74,75]. The disruption of the blood-brain barrier (BBB) and increased permeability of the compromised gastrointestinal tract facilitate the crossing of LPS and abnormal amyloids that may reach the CNS via the oronasal cavity [76].

The gut inflammation and dysbiosis of the intestinal-brain axis generally result in increased LPS release. They are responsible for triggering an inflammatory cascade with the production of pro-inflammatory mediators in the CNS that have the power to activate microglial cells and astrocytes and stimulate the NF-kB signaling pathway leading to neuronal death [77,78]. The effect of exotoxins like LPS over microglial cells can also impair amyloid beta clearance, leading to its accumulation and facilitating AD pathogenesis [79].

Gut microbiome dysbiosis can contribute to systemic inflammation, BBB breakdown, brain neuroinflammation, and neurodegeneration. Circulating TNF levels can promote endothelial permeability by reducing the endothelial glycocalyx and altering its thickness [80]. Microglia activation can stimulate astrocytes to secrete TNF and glutamate [81]. Intraperitoneal injections of LPS activated microglia in mice and impaired their capacity to synchronize local neural circuits and increased microglia traffic toward cerebral blood vessels, causing an increase in BBB permeability [82].

## 3. Effect of ApoE Mimetic Peptides on Intestinal and Brain Injuries

### 3.1. ApoE and ApoE Mimetic Peptides Biology

Apolipoprotein E (apoE = protein; APOE = gene) is a crucial chaperone molecule that transports hydrophobic cholesterol from cells to the liver for metabolization and clearance. ApoE is a 299-amino acid protein encoded by a single human polymorphic gene. The APOE gene on chromosome 19 carries the three most common alleles (APOE2, APOE3, and APOE4) that direct the synthesis of three protein isoforms, produced because of two-point mutations in the DNA sequence. These polymorphisms cause direct substitution at positions 112 and 158 of the apoE protein, where the apoE2 isoform contains cys112 and cys158, the apoE3 isoform contains cys112 and arg 158, and the apoE4 isoform contains arg 112 and arg 158 [83].

ApoE is synthesized mainly in the liver, brain, spleen, kidneys, adrenal gland, lungs, macrophage-monocytes, the central and peripheral nervous systems, and muscle tissues. It has an important anti-inflammatory function by reducing the synthesis of interleukin-2 (IL-2) and converting the pro-inflammatory macrophage M1 phenotype into M2, which has an anti-inflammatory action and an immunomodulatory response through T lymphocytes [84,85]. However, ApoE acts in neuronal recovery by controlling lipid homeostasis, metabolism, and transport of cholesterol and triglycerides [86,87]. The APOE4 allele appears to protect against enteric infections and malnutrition [88], effects that may be related to cholesterol shifting from the enteric pathogen to the host to improve the innate immune responses [89,90].

ApoE mimetic peptides are small peptides derived from the native receptor binding region (residues 130–150) of apoE, which contain at least one amphipathic helix. Like holo-apoE’s receptor-binding domain, these peptides can bind to apoE cell surface receptors and maintain the same functional activity as the intact protein. They are also called apomimetics, apomimetic peptides, apoE-derived peptides, or apolipoprotein-derived peptides. These peptides have the potential to improve the function of endogenous HDLs [91]. The 34 kDa ApoE protein typically exists as a dimer with two helical horseshoe configurations layered on top of one another. This structure contains a low-density lipoprotein receptor (LDLR) binding domain and a major lipid binding domain [92]. The amino terminus of apoE is composed of four amphipathic helices, with the LDLR binding domain located in helix number 4 [93]. ApoE mimetic peptides have been shown to bind to members of the LDLR family and thereby suppress inflammation and neurotoxicity and help mediate antiviral effects [94,95,96].

### 3.2. The Role of ApoE and ApoE Mimetic Peptides on the Intestinal Barrier, Microbiota and Inflammation

ApoE has essential functions in the metabolism of the lipid profile and is also related to changes in immune responses [97]. Studies using experimental animals that lack apoE have exhibited increased inflammatory responses in a sepsis model [98,99]. From amino acid residues located in the region of binding to the apoE holoprotein receptor, the mimetic peptide of this apolipoprotein, called COG133, was created. COG133 has been shown to reduce TNF and nitric oxide (NO) in LPS-stimulated microglial cells [94]. In addition, COG133 suppressed LPS-induced systemic and cerebral inflammatory responses in mice injected with LPS [95].

In 2008, the first study was published documenting the inhibitory effect of an apoE mimetic peptide on the NF-kB pathway in young adult mouse colon (YAMC) cells, which resemble colonic human epithelial cells [100]. This research was a pioneer in attributing apoE roles in colitis and other intestinal inflammatory conditions and opened new venues to investigate the potential benefits of these compounds against enteropathogens. The apoE-mimetic peptide COG112 has been shown to inhibit bacterial-induced expression of iNOS mRNA and protein and NO production, well-known players in colitis pathogenesis. Of note, iNOS has been shown to immunolocalize within the colonic epithelium during *Citrobacter rodentium* colitis [101]. ApoE mimetic peptides have been shown to modulate macrophage phenotypes, downregulating pro-inflammatory cytokines [102]. Hence, these findings support the idea that apoE mimetics could be a good target for therapeutic approaches to improve colitis.

One potential protective mechanism of apoE mimetic peptides is to replenish apoE circulating levels to restore homeostasis by increasing the levels of body “apoE-equivalents”. Native apoE protein has been found to display antibacterial effects, especially against Gram-negative bacteria such as *E. coli* [103,104]. Corroborating with this findings, apoE protein levels are lower in patients with sepsis [105]. Although this is a compelling possibility, the complex apoE-HDL or HDL alone (or other plasmatic lipoproteins) may also have a role in diminishing LPS [106,107]. Other biological interactions, such as those with the innate immune system, are still relatively unexplored and require further investigation.

There is great concern about the clinical relevance of studying the intestinal microbiota in patients with AD. Some studies showed that changes in intestinal microbial composition were associated with cognition, especially memory [108,109], while others collected important clinical characteristics, such as educational level, APOE genetic background, cognitive function, and illness severity. The occurrence of the genera *Saccharimonadales*, *Patescibacteria* and *Saccharimonadia* was found to be related to aging. In addition, populations of *Erysipelatoclostridiaceae*, *Erysipelotrichales*, *Saccharimonadales*, *Patescibacteria* and *Saccharimonadia* were associated with the APOE4 genotype [110]. APOE 4 has been shown to be a strong genetic risk factor for AD [111], with the risks of AD being increased by 3–4 times in APOE4 heterozygotes and 8–12 times in APOE4/4 homozygotes [112]. Tran and colleagues found an association between APOE genotype and gut microbiota in human and transgenic mice [113]. The strong genetic association between APOE and AD [114,115] could be reflected by an altered intestinal microbiota harboring bacteria taxon that favor AD pathogenesis. Recent data support a relationship between APOE4 status, the gut microbiome, and the neuropathology of AD [110,116]. The mechanisms by which APOE alleles influence the gut microbiome remain unclear. However, APOE4 has been associated with an increased inflammatory response to LPS, an endotoxin presents in all gram-negative bacteria, both in humans and in mice [95,117].

Interestingly, APOE4-targeted replacement (TR) mice are more resistant to *Cryptosporidium* intestinal infection than APOE3 mice [88]. The APOE4 allele in humans has been associated with better neuropsychological outcomes in children afflicted with diarrheal diseases early in life in developing countries [90,118]. Intriguing is the finding that APOE4 is associated with low c-reactive protein blood levels and pro-inflammatory innate responses in gather-hunter populations living in remote areas of the Amazon [119,120] under highly contaminated environments. APOE4 is now being considered a gene candidate for antagonistic pleiotropy over human evolutionary changes, and enteric helminths may be the strongest driving force for keeping APOE4 in our genetic pool despite its deleterious effects during aging under modern western lifestyles [106,121]. Infectious diseases and famine have been recognized as strong driving forces to shape our genome during human evolution and the modernity era [122,123]. A better understanding of the role of APOE polymorphisms on the gut microbiome may provide new approaches to combating the deleterious genetic effects of APOE on human diseases.

ApoE4 knock-in males also showed the most prominent alterations in the gut microbiome involved in inflammation and energy deprivation [124]. Changes in their gut microbiota include an increase in well-established human AD microbiota markers (*Akkermansia muciniphila* and *Prevotella* spp.) [125]. This effect was found along with a reduction in IgG/IgA-producing *Bacteroide ovatus* (decreased serum IgG/IgA have been found among AD patients) and increases in several pro-inflammatory taxa [126].

A Brazilian cohort of shantytown children, under active surveillance for malnutrition and diarrhea in their first years of life bearing the APOE4 allele, showed later improved growth and cognitive outcomes following glutamine and vitamin A supplementation [127]. These findings need to be taken with caution and confirmed with larger cohort studies. Gut-trophic nutrient supplementation may be particularly important in children living in adverse environments to reduce intestinal inflammation caused by enteric diseases, with improvements in gut absorptive surface area and thus key nutrient bioavailability to the brain.

Signaling pathways and changes involved in the pathophysiology of cancer and during its treatment are essential for identifying pharmacological targets capable of reducing the uncontrolled growth of neoplastic cells. Intestinal mucositis is one of the main side effects of 5-FU, which is a chemotherapy treatment for colorectal cancer. In this context, it was demonstrated that apoE exerts anti-inflammatory effects on intestinal mucositis in animal models. Results found in intestinal epithelial cells (IEC-18) suggested a protective role for an apolipoprotein E mimetic peptide, COG1410, after 5-FU challenge through indirect activation of the Wnt/β-catenin pathway [128]. Treatment with apoE peptide COG 133 in IEC-6 after 5-FU reduces gene expression of the β-catenin destruction complex, which restricts Wnt canonical signaling, leading to increase cell viability, hence showing protective effects on intestinal cell monolayers [129].

Table 1 depicts selected literature of apoE mimetic peptides, highlighting their roles on the gut-brain axis.

### 3.3. The Role of ApoE and ApoE Mimetic Peptides in the Blood-Brain Barrier and Neuroprotection

ApoE is abundantly found in the CNS, being involved in many brain functions. It is mainly produced by glial cells, and once released, apoE may be actively engaged in dynamic plastic functions, such as synaptogenesis, neurogenesis, and myelination [130,131,132]. Thus, it is not surprising that the interest in investigating apoE’s beneficial roles in brain disorders has increased. However, since the entire 299-amino acid apoE protein could not cross the BBB due to its large size, apoE mimetic peptides that mimic the function of the native apoE, can be used instead and are translocated into the brain to exert their effects [95,133].

The BBB is a highly specialized structure that simultaneously protects the brain against potentially harmful external agents and ensures the correct supply of nutrients and oxygen. It is composed by brain endothelial cells (ECs) lining the cerebral blood vessels that together with pericytes, basement membrane, astrocytes, microglia, and neurons form the neurovascular unit (NVU). Brain capillary ECs restrict molecules trafficking in between the blood and the brain. The physical barrier nature of the BBB and its selective permeability are due to inter-endothelial tight and adheres junctions that comprise transmembrane and cytoplasmic scaffolding proteins, such as occludin, claudin-5, zonula occludens-1 (ZO-1), VE-cadherin, and β-catenin, conferring the low paracellular permeability. Furthermore, normal brain endothelium lacks fenestrae and has limited vesicular transport (transcytosis). The uniqueness of the BBB endothelium is also endorsed by the abundant expression of selective influx and efflux transporters, and receptors. The elevated number of mitochondria in ECs reflects a high energy demand for active ATP-dependent transport, and these cells express several transporters that aid in lowering nutrients down to their concentration gradients [134].

Taking into consideration the critical role of the BBB for proper brain function, this barrier has been an important topic of investigation in several brain pathologies, including those that are not traditionally identified as neurovascular disorders. It is now unquestionable that neurodegenerative disorders, such as Alzheimer’s disease and amyotrophic lateral sclerosis (ALS), are also associated with defective BBB function [135]. Interestingly, human apoE, particularly APOE4, was identified as a major genetic risk factor for Alzheimer’s disease by differential effects on amyloid-β accumulation in the brain and its vasculature, as well as for poor neurological outcome after traumatic brain injury and hemorrhage [136,137]. In fact, both normal APOE4 carriers and individuals with APOE4-associated disorders have neurovascular dysfunction [135]. Additionally, APOE4 carriers present altered cerebral blood flow (CBF) [138] that is recognized to be strongly associated with cognitive deficits, including language and verbal memory [139]. Bell and colleagues also demonstrated that APOE2, APOE3, and murine ApoE support BBB integrity, whereas APOE4 has the opposite effect. The authors further identified the proinflammatory cytokine CypA, and specifically the CypA–NF-kB–MMP9 pathway in pericytes, as a key pathway in APOE4-induced BBB breakdown [137]. In accordance with these observations, Lin and colleagues proved that APOE4 transgenic mice have CBF reductions, BBB impairments and learning deficits [140].

Moreover, rapamycin was able to restore brain vascular functions in young APOE4 mice, which was associated with the reduced CypA levels in the vasculature, a proinflammatory cytokine known to cause BBB breakdown by activating the NF-κB-MMP9 pathway as previously mentioned [137,141]. A recent study using conditionally humanized APOE knock-in mice also showed increased BBB permeability in the APOE4 mice but not in the APOE3 or APOE2 mice, proving once again the importance of MMP9 in ApoE-dependent BBB deficits [142]. Curiously, BBB leakage was rescued following the knock-out of apoE4 from astrocytes. The authors also stated that full APOE knockout of all apoE protein from birth presents BBB leakage suggesting multiple mechanisms by which apoE protein (or its absence) influences the integrity of the BBB [127]. In fact, the lack of apoE has been previously associated with BBB breakdown, increased BBB susceptibility to injury, and exacerbation of brain edema after brain trauma, explaining the cognitive impairment presented by apoE-deficient mice after closed head injury [143]. Data also suggest that apoE deficiency is directly related to higher severity of early brain injury followed by poor outcomes after experimental SAH by rapidly increasing BBB permeability and disruption [144]. Specifically, the severe BBB leakage in APOE^−/−^ mice was also associated with a reduction of pericytes and endothelial tight junctions [145].

Literature has shed light on the risk and protective functions of different apoE isoforms differ considerably. Thus, the design of apoE mimetic peptides of protective isoforms seems to be a promising approach in CNS disorders. In recent years, studies have demonstrated that apoE mimetic peptides have played a beneficial role in different experimental models by modulating the expression of pro-inflammatory cytokines in the body and the brain [106].

These peptides have been shown to be efficient in attenuating BBB disruption upon brain injury and subarachnoid hemorrhage, mediating neuroprotection, and reducing BBB permeability, where their effects seem to be linked to the activation of the CypA-NF-κB-MMP-9 pathway [146]. ApoE mimetic peptides function to abrogate the systemic and brain-specific acute inflammatory response when administered to mice previously injected with LPS and reduce their brain levels of TNF and IL-6 [95]. In mice, the ApoE mimetic peptide COG1410 reduces early brain injury after experimental subarachnoid hemorrhage (SAH) by attenuating blood-brain barrier disruption, causing less tight junction protein breakdown and vacuolization between endothelial cells, resulting in reduced endothelial cell apoptosis [146].

The apoE-derived mimetic peptide COG1410 attenuates early acute brain injury by lessening neuronal apoptosis, microglia activation, and BBB leakage, resulting in an increase in cerebral glucose uptake and promoting attenuation of neurocognitive dysfunction in mice subjected to a subarachnoid hemorrhage model [144]. COG1410 showed similar neuroprotective effects in a traumatic brain injury (TBI) mouse model, with increased cerebral glucose uptake likely due to the preservation of BBB integrity. It enhanced blood flow, suggesting that this apoE-derived mimetic peptide can regulate the expression of the glucose transporter-4 (Glut-4) [147]. In addition, COG1410 ameliorates both the TBI lesion volume and vasogenic edema, which are linked to the suppression of MMP-9 activity [148]. Additionally, COG1410 facilitates neuronal survival and autophagy by indirectly phosphorylating GSK-3b, an important event that regulates autophagy in the early brain injury model, which could represent a mechanism to attenuate the acute brain insult caused by subarachnoid hemorrhage [149]. The apoE mimetic peptide COG1410 also downregulated the pro-apoptotic proteins BAX and cleaved caspase 3 and upregulated the expression of BCL2, anti-apoptotic protein, decreasing endothelial cell death and promoting BBB integrity likely by activating the Akt pathway and suppressing the JNK/c-Jun pathway [91,150]. CN-105 is another apoE-derived mimic peptide involved in many preclinical CNS injury studies with positive neuroprotective results and safety [151]. Altogether, these studies suggest that apoE mimetic peptides could be seen as a potential neuroprotective therapeutic tool to decrease neuronal death and attenuate BBB disruption and neuroinflammation, promoting functional recovery after brain injury.

ApoE plays an essential role in acute brain injury and neuronal recovery post-insult. However, the specific mechanisms behind it have yet to be clarified. The low-density lipoprotein receptor-related protein 1 (LRP-1) or apoE receptor is present in microglia and neurons and has been pointed out as a critical factor for the anti-inflammatory and neuroprotective effects mediated by apoE [152].

Therefore, apoE mimetic peptides that hold this specific receptor-binding site seem to bind LRP-1 in neurons affecting N-methyl-aspartic acid (NMDA) receptors, leading to a cellular response like that with apoE full-length protein [153]. In primary glial and neuronal cell lines, the apoE mimetic peptide COG133 was reported to interact with LRP-1 to inhibit NMDA receptors, decreasing NMDA-mediated calcium excitotoxicity and neuronal death [147,149]. In an experimental model of subarachnoid hemorrhage (SAH) in mice, intravenously injected COG133 resulted in improved BBB function by inhibiting the CypA-NF-κB-MMP9 pathway and reduced neuronal pyroptosis by actively suppressing NLRP3 inflammasome cascade activation [151,154]. Additionally, apoE mimetic peptides can be associated with nanoparticles to facilitate the entrance of hydrophobic molecules into the brain and exert their beneficial effects, which was shown in a pre-clinical model of medulloblastoma [155].

In another murine SAH study, the apoE mimetic peptide CN-105 was able to reduce cerebral vasospasm with lasting improvements in histological and functional results due to its anti-inflammatory effect. This probably occurred due to a reduction in the hippocampus of FJB-positive cells, microgliosis, and an increase in the number of NeuN-positive cells [156].

Authors have suggested a mechanism where apoE mimetic peptides modulate positively on protein phosphatase 2A (PP2A) activity via an antagonistic action on SET binding, which leads to reduced inflammatory response signaling by decreasing activation levels of phosphorylated signaling kinases. This mechanism occurs when apoE mimetic peptides and apoE bind to the C-terminal part of the SET protein, which plays an important role in the physiological inhibition of PP2A, leading to increased PP2A-mediated phosphatase activity. The increase in PP2A activity reduces the activation of inflammatory signaling pathways such as TLR-4 pathways with LPS, MAP kinases, and NF-kB, as evidenced by the reduced levels of phosphorylated p38-MAP kinase and Akt kinase, as well as the reduced release of nitric oxide [157].

**Table 1:** Summary of apoE mimetic peptide research findings in inflammatory gut and brain models.

Authors	ApoE Mimetic Peptides	Effects on the Small Intestine or Brain/Blood Brain Barrier	Mechanism of Action
Laskowitz et al. [94]	COG133	Suppresses inflammatory responses	Inhibits the production of TNF and nitric oxide stimulated by LPS in microglial cells of ApoE-deficient mouse pups
Lynch et al. [95]	COG133	Modifies systemic and cerebral inflammatory responses	Cerebral and systemic elevations of TNF and IL-6 were identified in APOE4-TR mice after intravenous LPS injection. Improved expression of these cytokines when treated with the mimetic peptide
Azevedo et al. [96]	COG 133	Displays anti-inflammatory effects	Recovery of viability and migration of intestinal cells, reduction of intestinal myeloperoxidase levels and expression of IL-1β, TNF, iNOS mRNAs with improvement in IL-10 in a model of intestinal mucositis in mice
Singh et al. [100]	COG112	Attenuates colon inflammation	Inhibits bacterial-induced expression of iNOS mRNA and protein and NO production in colonic epithelial cells using rodent model studies
Pessoa et al. [128]	COG1410	Improves wound healing of IEC-18 monolayers	Restores intestinal IEC-18 cell monolayer after 5-FU injury through indirect activation of the Wnt/β-catenin pathway
Pessoa et al. [129]	COG133	Improves wound healing of IEC-6 monolayers	Injury of intestinal IEC-6 cells by 5-FU resulted in decreased levels of APC transcript and Wnt/β-catenin pathway, which were recovered with mimetic peptide treatment
Wu et al. [144]	COG1410	Attenuating tissue damage and reducing inflammation	Lessens neuronal apoptosis, microglia activation, and BBB leakage resulting in an increase of cerebral glucose uptake and promoting neurocognitive dysfunction attenuation in mice subjected to a subarachnoid hemorrhage model
Pang et al. [146]	COG1410	Reduces early brain injury	Attenuates BBB disruption, causing less tight junction breakdown and vacuolization between endothelial cells, less endothelial cell apoptosis, in an experimental subarachnoid hemorrhage (SAH) model in mice
Qin et al. [147]	COG1410	Displays neuroprotective effects	Increases cerebral glucose uptake with recovery of vestibulomotor deficits associated with rescued BBB
Li et al. [149]	COG1410	Displays neuroprotective effects	In a model of neuronal autophagy after SAH, affecting the phosphorylation of GSK-3β in neuronal and brain tissue, resulting in an improvement in the neurological score
Wang et al. [153]	COG1410	Attenuates tissue damage and reducing inflammation	Reduces cerebral edema, inflammation, and infarct area, also decreasing TNF RNA expression using a murine model of transient focal cerebral ischemia and reperfusion.
Zhang et al. [154]	COG133	Plays a neuroprotective role by protecting blood-brain barrier function and inhibiting brain cell pyroptosis	Inhibits blood-brain barrier impairment through the proinflammatory CypA-NF-κB-MMP9 pathway and reduces neuronal pyroptosis by inhibiting NLRP3 inflammasome activation.
Liu et al. [156]	CN-105	Reduces cerebral vasospasm and anti-inflammatory effect	Likely due to a reduction of FJB-positive cells, microgliosis, and an increase in the number of NeuN-positive cells in the hippocampus
Christensen et al. [157]	COG1410	Reduces inflammatory response	Modulates positively protein phosphatase 2A (PP2A) activity, with an antagonistic action on SET binding, signaling by decreasing activation levels of phosphorylated signaling kinases
Zhao et al. [158]	COG1410	Protects against brain injury-induced apoptosis and recovers learning and memory function	Improvements via extracellular signal-regulated mitochondrial apoptotic mechanism and Bax kinase1/2-Bax, attenuating the apoptotic signaling pathway by downregulating Bax protein and cytochrome C expression

**Figure 2:**
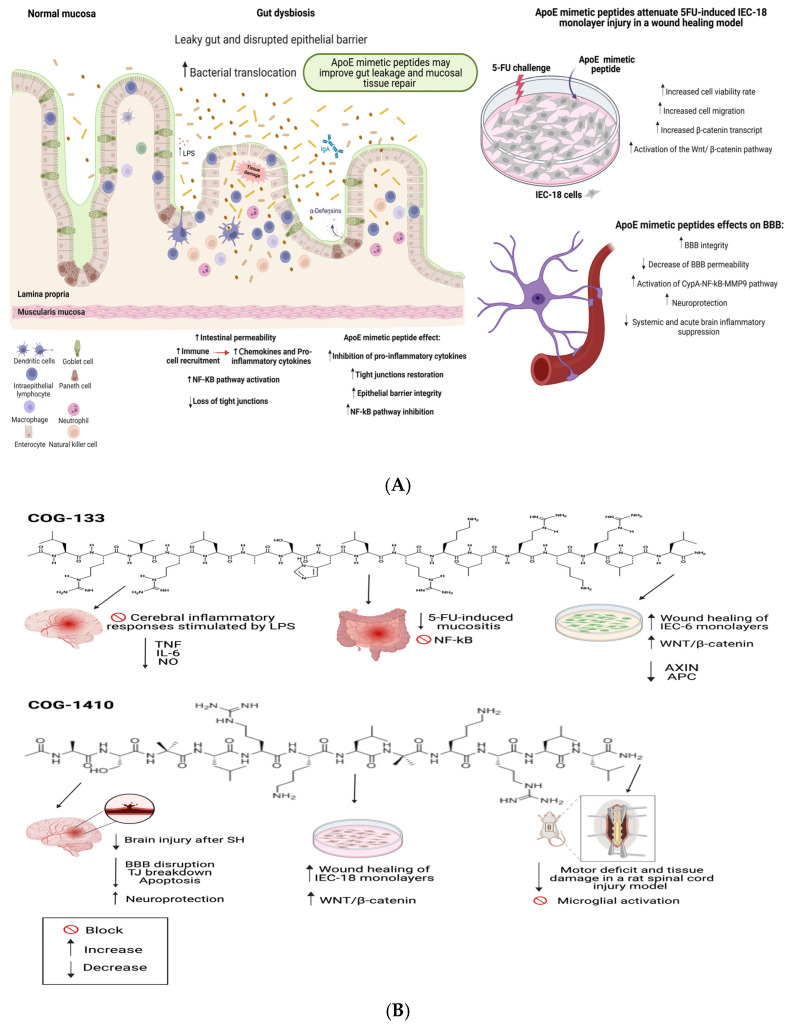
(**A**) Selected ApoE mimetic peptides (COG 133 and COG144) with beneficial effects in different models of intestinal and CNS injury. (**B**) ApoE mimetic peptides could improve IEC-6 and IEC-18 (murine crypt intestinal cell line) cell monolayer recovery with more Wnt-βcatenin and less β-catenin degradation complex (AXIN and APC) mRNA transcription and intestinal mucositis-driven inflammation following 5-Fluorouracil (5-FU) challenge. TJ = tight junctions, BBB = blood-brain barrier, SH = stroke hemorrhage. APC = adenomatous polyposis coli. Arrow up = increase and arrow down = decrease. Figure created with BioRender.com.

**Figure 3:**
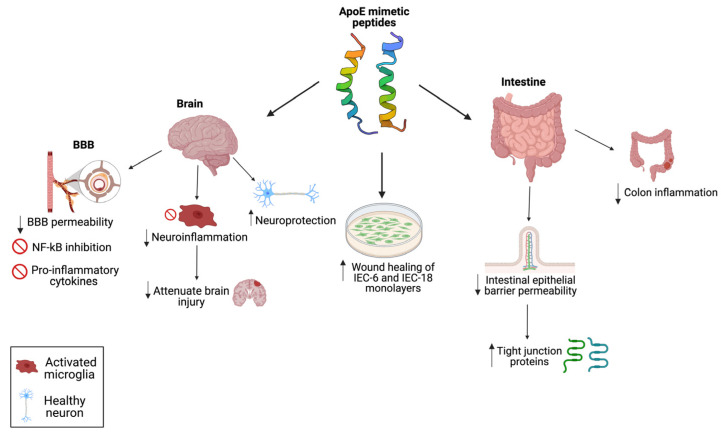
Schematic outline of the most beneficial effects of the apoE mimetic peptides based on preclinical models to support the potential use of these compounds to remediate the environmental enteric dysfunction-related outcomes. Arrow up = increase and arrow down = decrease. Figure created with BioRender.com.

Figure 2 and Figure 3 summarize the potential intestinal and brain protective mechanisms of apoE peptides, regulating the gut-brain axis, in different experimental models.

In a study in which the authors administered the apoE mimetic peptide in the caudal vein of mice with diffuse brain injury before and after injury, they suggested that the apoE mimetic peptides protect against brain injury-induced apoptosis and more rapidly recover learning and memory functions. This occurs via the extracellular signal-regulated mitochondrial apoptotic mechanism Bax kinase1/2-Bax. ApoE-mimetic peptide attenuates the apoptotic signaling pathway by downregulating Bax protein and cytochrome C expression after brain injury, reducing extracellular signal-regulated kinase1/2 phosphorylation, decreasing the amount of malondialdehyde, and increasing the activity of superoxide dismutase in a dose-dependent manner [158].

These studies suggest that apoE mimetic peptides could be seen as a potential neuroprotective therapeutic tool to decrease neuronal death and attenuate BBB disruption and neuroinflammation, promoting functional recovery after brain injury. In sum, apoE-mimetic peptides are promising therapeutic resources in preclinical and clinical settings as candidates for treating various CNS disorders.

Further evidence is needed on the possible direct effects of apoE and the apoE-mimetic peptides on the function and integrity of the BBB. Novel BBB organoids are now available [159] and could be generated from undernourished mice to dissect these effects.

**Figure 4:**
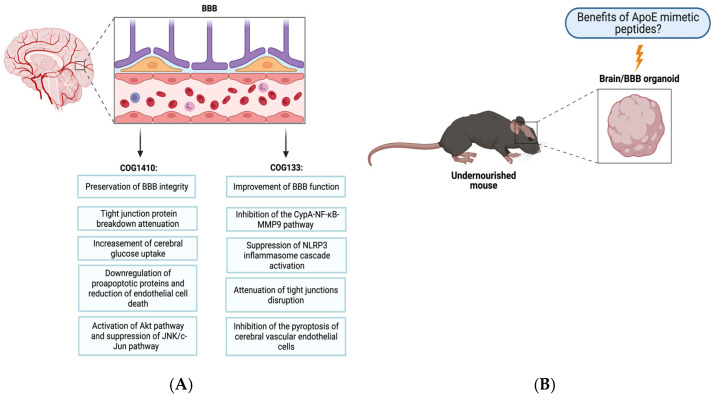
(**A**) Summary of apoE mimetic peptides beneficial effects on the blood-brain barrier (BBB) in models of brain injury. (**B**) The assessment of novel in vitro models of BBB and brain organoids may help to elucidate whether selected apoE peptides could be beneficial in preserving the gut-brain axis of mice afflicted with malnutrition/enteric infections. Figure created with BioRender.com.

Figure 4 shows what is currently known about apoE peptides’ role in protecting the BBB and the potential use of organoid models to assess the mechanisms and biological functions of these compounds in laboratory mice challenged with enteric infections and malnutrition.

## 4. Conclusions

ApoE mimetic peptides are promising compounds in a multi-faceted strategy approach to break the vicious cycle of malnutrition and enteric diseases, along with antimicrobials, potable water, micronutrients, vitamins, and even easy-in-place handwashing and other hygiene measures to mitigate EED in children. Such a condition may chronically and subclinically afflict children exposed to enteric pathogens in impoverished settings in developing areas leading to poor growth and developmental trajectories. The environmental enteric dysfunction of a subclinical course may be elusive to public health authorities and might be neglected with long-term effects including potentially increased morbidity with aging. Any successful intervention strategy to alleviate this problem should be balanced in cost-benefit and complement other strategies to provide effective solutions for people in greatest need.

Screening and testing of novel apoE mimetic peptides with antibacterial effects in the preclinical setting are warranted to seek EED amelioration, perhaps reducing the use of widespread and indiscriminate antibiotics [160,161]. Large and well-designed multicenter cohort studies are desirable to test whether these peptides may play a role in reducing intestinal barrier leakage and improving the healthy microbiota, perhaps with glutamine and zinc, thus reducing diarrhea and enteric disease rates (even without overt diarrhea) in children early in life, which are critical times of brain development. In addition, such peptides may synergically benefit the gut-brain axis by enhancing the integrity of intercellular junctions in the intestinal and blood-brain barriers, reducing the entrance into the brain of gut-to-blood circulating bacteria or LPS, and reducing neutrophil-related inflammatory products that may lead to prolonged brain neuroinflammation and neurologic deficits.

## Data Availability

Not applicable.
